# Ganoderma Lucidum Triterpenoids Improve Maternal Separation-Induced Anxiety- and Depression-like Behaviors in Mice by Mitigating Inflammation in the Periphery and Brain

**DOI:** 10.3390/nu14112268

**Published:** 2022-05-28

**Authors:** Xue Mi, Gui-Rong Zeng, Jie-Qing Liu, Zhou-Song Luo, Ling Zhang, Xiao-Man Dai, Wen-Ting Fang, Jing Zhang, Xiao-Chun Chen

**Affiliations:** 1 Fujian Key Laboratory of Molecular Neurology, Institute of Neuroscience, Fujian Medical University, Fuzhou 350004, China; mixue@fjmu.edu.cn (X.M.); zenggr99@163.com (G.-R.Z.); zhsluo@fjmu.edu.cn (Z.-S.L.); sdytdaixiaomanld@163.com (X.-M.D.); drwenting@163.com (W.-T.F.); 2 School of Medicine, Huaqiao University, Quanzhou 362021, China; liujieqing@hqu.edu.cn (J.-Q.L.); zl15759683078@163.com (L.Z.); 3 Department of Neurology and Geriatrics, Fujian Institute of Geriatrics, Fujian Medical University Union Hospital, Fuzhou 350001, China

**Keywords:** maternal separation, Ganoderma lucidum triterpenoids, anxiety, depression, inflammatory response

## Abstract

Although early life stress (ELS) can increase susceptibility to adulthood psychiatric disorders and produce a greater inflammatory response in a stressful event, targeted preventive and therapeutic drugs still remain scarce. Ganoderma lucidum triterpenoids (GLTs) can exert anti-inflammatory effects in the periphery and central nervous systems. This study employed a combined model of “childhood maternal separation + adulthood sub-stress” to explore whether GLTs may alleviate anxiety- and depression-like behaviors in male and female mice by mitigating inflammation. Male and female pups were separated from their mothers for four hours per day from postnatal day 1 (PND 1) to PND 21; starting from PND 56, GLTs were administered intraperitoneally once daily for three weeks and followed by three days of sub-stress. Results showed that maternal separation increased the anxiety- and depression-like behaviors in both male and female mice, which disappeared after the preemptive GLTs treatment (40 mg/kg) before adulthood sub-stress. Maternal separation up-regulated the pro-inflammatory markers in the periphery and brain, and activated microglia in the prefrontal cortex and hippocampus. All the abnormalities were reversed by GLTs administration, with no adverse effects on immune organ indices, liver, and renal function. Our findings suggest that GLTs can be a promising candidate in treating ELS-induced psychiatric disorders.

## 1. Introduction

Major depression is an urgent and growing public health threat. Survey data from the World Health Organization indicate that childhood adversities are highly prevalent, with maladaptive family environment (e.g., child abuse, neglect) strongly associated with adult psychopathology [1]. Other epidemiological evidence also documented that the early life is a period of vulnerability and sensitivity, and episodes of early life stress (ELS; e.g., traumatic events, violence exposure, child abuse, and negligence) will raise the likelihood of neuropsychiatric abnormalities in the later life, such as mood and anxiety disorders [2,3,4,5].

Experiences of childhood adversity are continually associated with greater inflammation in adulthood. A life-course study pinpointed childhood maltreatment as an independent risk for adulthood inflammation in a significantly correlated dose-response manner [6]. According to the available literature, male major depression patients who experienced childhood adversities display an aggravated proinflammatory response to psychosocial stresses, signifying a link between major depression, childhood adversities, and adverse inflammation-associated health outcomes [7]. For individuals growing up in a harsher family environment with a negative emotionality, a greater concentration of interleukin 6 (IL-6) may surface when they experience stressful life events compared with individuals brought up in an amiable, harmonious family environment with a positive emotionality [8]. Other studies demonstrate that a childhood exposure to an environment characterized by a low socioeconomic status and harsh unpredictability appears to increase the circulating inflammatory marker C-reactive protein in adulthood [9]. Altogether, these pieces of evidence demonstrate that experiences of childhood adversities will aggravate the proinflammatory response when the individuals encounter stressful events in adulthood.

As a medicinal mushroom, *Ganoderma lucidum* (*G. lucidum*) plays a significant role in disease treatments and longevity promotion in several countries, especially in the East Asian [10]. As a category of the main bioactive and medicative components in *G. lucidum*, Ganoderma lucidum triterpenoids (GLTs) are documented to perform various pharmacological actions such as anticancer [11], anti-inflammation [12,13], anti-atherosclerosis [14], anti-hyperlipidemia [15], antioxidants [16,17], antivirus [18], hepatoprotection [19], retardation renal cyst [20], renal fibrosis development, and protection of renal ischemia-reperfusion injury [21]. Moreover, recent studies evidence that GLTs can also exert pharmacological effects on the nervous system, alleviating neuronal apoptosis [22] and neuroinflammation [23] in Alzheimer’s disease mice. As components of GLTs, ganoderic acid [24] and deacetyl ganoderic acid F [25] were found respectively to improve 5-fluorouracil-induced cognitive dysfunction and inhibit lipopolysaccharide (LPS)-induced neural inflammation. However, the role of GLTs in ELS-induced anxiety and depression has largely remained unelucidated.

In this study, we investigated whether GLTs would, via the inhibition of inflammation, mitigate the anxiety- and depression-like behaviors in the male and female mice that experienced maternal separation and adulthood sub-stress. The results showed that the preemptive GLTs intervention decreased the inflammatory response in the brain and the periphery, and ameliorated the anxiety- and depression-like behaviors induced by maternal separation and adulthood sub-stress. Additionally, GLTs produced no toxic effects on immune organs, the liver, and kidney function in mice. The findings illustrate that GLTs can serve as a therapeutic candidate in treating ELS-induced anxiety and depression.

## 2. Materials and Methods

### 2.1. Extraction and Isolation

The fruiting bodies of *G. lucidum* (30 kg) were obtained from Wuyishan Ganoderma Lucidum Planting Base (Wutun Town, Wuyishan City, Nanping City, Fujian, China. Cultivated variety: GL-8). A voucher specimen (2016101) was preserved in our laboratory.

The fruiting bodies were dried in shade conditions, pulverized to coarse powder, and then extracted with 95% ethanol (50 L) at room temperature. Ethanol was recovered by distillation under reduced pressure, and the black extract was attained. The extract was resuspended in water and successively partitioned with ethyl acetate four times. The ethyl acetate layer was collected and combined and concentrated to dryness, obtaining 0.9 kg of ethyl acetate extract for separation. The obtained ethyl acetate extract was separated by D101 macroporous resin to remove the pigment and further eluted with methanol/H_2_O (MeOH/H_2_O, 50:50, 70:30, 80:20, 100:1, *v*/*v*), producing four fractions (Fr.1–Fr.4). The Fr.1 fraction (250 g) was subjected to silica gel CC (15 × 180 cm) eluted with methylene chloride/methanol (100:1, 85:1, 50:1, 20:1, and 5:1, *v*/*v*) and resulted in eight fractions (Fr.1.1–Fr.1.8) according to silica gel TLC analyses. Subsequently, the Fr.1.6 fraction (29 g) was separated to three fractions (Fr.1.6.1–Fr.1.6.3) on a silica gel CC (6 × 40 cm) with methylene chloride/methanol (100:1, 50:1, 20:1, and 5:1, *v*/*v*) as eluent. Fr.1.6.3 (14.9 g) was used for liquid chromatography–mass spectrometry (LC-MS) and subsequent drug administration in animals. The data obtained by LC-MS were matched with the Global Natural Products Social Molecular Network (GNPS) to visualize the correlation of molecules in the mixture, which revealed that the mixture contained a large amount of Ganoderma lucidum triterpenoids with similar skeletons, resulting in 17 inferred triterpenoids. Detailed analysis results can be found in Supplementary Material Document 1. The drug was solubilized in a compound solution (2% DMSO + 10% PEG300 + 2% Tween 80 + 86% saline), and the compound solution without the drug was treated as vehicle control.

### 2.2. Animals

A total of 36 adult C57BL/6J mice (24 females and 12 males) were provided by Zhejiang Vital River Laboratory Animal Technology Co., Ltd. (Certificate No. SCXK 2019-0001, Pinghu, Zhejiang, China). All animals were raised in standard housing conditions (12/12 h light/dark cycle, lights on at 7:00 am; a controlled temperature of 22 ± 1 °C, and a humidity of 50 ± 10%) with food and water ad libitum except during behavioral tests. Animals were given one week to acclimatize to the new environment before the behavioral testing. Maximal care was taken to reduce animal suffering and the number of animals used. All experimental animal protocols were approved by the Ethical Committee of Institutional Animal Care and Use of Fujian Medical University (FJMU ACUC 2021-0399). The procedures were performed followed the European Community Guidelines for the Care and Use of Experimental Animals (Directive 2010/63/EU).

### 2.3. Experimental Protocol

The overall experimental procedures for this study are briefly explained as follows. After delivery, dams were randomly assigned to the control and maternally separated (MS) group. The latter was subjected to both maternal separation and limited nesting material [26]. The pups were separated from dams for 4 h per day during PND 1–21 with limited nesting material while the control littles were reared in standard circumstances. All the pups were weaned at PND 21 and housed by sex and raised under standard conditions. GLTs or vehicle treatment was initiated for all mice at eight weeks of age (56 days) for a total of three weeks. Subsequently, all the mice were subjected to sub-threshold variable stress (STVS) for three consecutive days and were individually housed after the final stressor. Behavioral tests were performed on the next day, and then all the mice were sacrificed at the end of the study. After a complete isoflurane anesthesia, the peripheral blood samples and relevant organs, such as spleens, brains, thymus, were collected for analysis. The organ mass indices were measured with the following formula: 10 × mean organ weight (mg)/mean body weight (g).

#### 2.3.1. Maternal Separation

Maternal separation was performed following a previous method with modifications [26]. At the age of eight weeks, one male mouse was mated with two nulliparous C57BL/6J female animals, and the delivery date was estimated at 20–22 days from the first mating day. Five days after mating, the males were removed, and the pregnant females were individually housed at least three days before the estimated delivery date and stayed in the cage with their own pups after birth. Litters were counted and weighed and transferred to clean cages on the day of birth (PND 0). A litter size of less than three or greater than nine was excluded from the experiment in order to ascertain sufficient nutrient supply and maternal care. Dams and their pups were assigned randomly to the MS or control group. The MS littles were separated from home cages and placed into plastic chambers housed inside a constant-temperature incubator (32–34 °C) for 4 h per day (from 10:00 am to 14:00 pm) from PND 1 to PND 21. The dams were then transferred to clean cages. All littles and dams were reunited in their original cages after 4 h of separation. During the period of separation, the dams were given limited nesting material (down to 1/3 of the standard need). Pups in the control group were reared under a standard animal facility until weaning. Cages of all pups were changed with minimal disruption every week, and the food and water were changed every three days. All the pups were weaned at PND 21 and housed by sex, with littermates grouped together, except for combining pups from different litters to maintain a size of three–five mice/cage.

#### 2.3.2. Sub-Threshold Variable Stress

The STVS was modified from a previously reported procedure [27]. Briefly, the STVS consisted of forced swimming in icy water (three times with a one-hour interval in between), tail suspension (for 1 h), and restraint (in a 50 mL conical tube for 1 h). In order to induce adulthood stress, all the mice were subjected to the three tests of the STVS separately in three consecutive days. Mice were housed individually following the final stressor, and behavioral tests were initiated the next day.

#### 2.3.3. Drug Administration

In order to ascertain the potential distinct effects of different GLTs doses on the mice of a specific sex, a total of 68 male and 74 female pups were raised into adulthood (8 weeks old) under standard conditions and enrolled in the study. They were respectively assigned in a random manner to six groups of 11–13 mice per group: Control + vehicle group; Control + GLTs (40 mg/kg) group; MS + vehicle group; MS + GLTs group, receiving GLTs at a dose of 10 or 20 or 40 mg/kg, respectively. The Control + GLTs and MS + GLTs groups received daily intraperitoneal (i.p.) injections of GLTs at 10 or 20 or 40 mg/kg for three weeks. The mice in Control + vehicle and MS + vehicle groups received the vehicle in order to emulate injection stress and eliminate the influence of the vehicle. The course of GLTs treatment was 3 weeks. The determination of the course was based on the actual emotional state of the mice after GLTs treatment. (We observed an increase in the spontaneous locomotion and enhanced activity since the third week of GLTs treatment. Therefore, the GLTs treatment was stopped at the end of the third week before proceeding with the next experiment.)

### 2.4. Behavioral Tests

Experimenters were blinded to both the grouping and drug administration of the mice, and the order of testing was counterbalanced. Before every test, all the mice were placed in the behavioral testing room and allowed to acclimate to the testing environment for at least 1 h. The behavioral tests were performed separately with an interval of 1–2 days.

#### 2.4.1. Open Field Test (OFT)

The OFT was performed as reported previously with slight modification [28]. Opaque plastic open field arenas (44 × 44 × 40 cm) were evenly illuminated in a dim light in a low-noise environment. The area of the arena was divided into a central square of 22 × 22 cm, four corners of 11 × 11 cm, and the other zones. Each mouse was placed in a corner and allowed to explore the open field arenas freely for 10 min. The locomotor activity and the time spent in the central and corner zones were recorded with the SuperMaze software (version 3.0; Shanghai Xinruan Information Tech Co., Shanghai, China). Less time spent in the central zone or more time spent in the corner zones was indicative of anxiety-like behaviors.

#### 2.4.2. Elevated plus Maze (EPM)

The EPM was conducted according to a previous method [29] which was placed 40 cm above the floor and comprised of two enclosed arms (30 × 5 × 15 cm) and two open arms (30 × 5 cm). Each mouse was left at the intersection of the four arms of the maze (5 × 5 cm), facing an open arm, and allowed to explore the arms freely for five minutes. Entries of and duration on each arm were recorded and automatically evaluated with SuperMaze software 3.0. After each test, the maze was cleaned with 75% ethanol. A decrease in duration or entries on the open arms indicated anxiety-like behaviors.

#### 2.4.3. Splash Test

According to available literature [26,30], the splash test was proceeded in a standard empty mouse cage under dim red light. The dorsal section of the mice was sprayed twice with 10% sucrose solution. Because of the viscosity of sucrose solution, the grooming behavior was initiated (i.e., the cleaning of the fur by licking or scratching), and the total grooming time was manually video recorded for five minutes by a blinded experimenter. A decrease in total grooming time was indicative of anxiety- or depression-like behaviors.

#### 2.4.4. Sucrose Preference Test (SPT)

Sucrose preference was performed as a measurement of the anhedonia-like behaviors in the mice using the previously reported method [31]. The animals were caged individually and supplied with two 50 mL conical tubes with spouted rubber tops filled with drink water overnight for habituation. Then, water in one of the conical tubes was substituted with a 1% sucrose solution (V900116, Sigma-Aldrich, Tokyo, Japan). Both bottles were weighed and switched every twelve hours to prevent location habituation. At the end of the 24 h testing, sucrose preference was measured by dividing the sucrose consumption by the total volume of liquid intake over the 24 h period. Decreased sucrose preference was an indicator of depression-like behaviors.

#### 2.4.5. Forced Swimming Test (FST)

The FST was conducted according to previous protocols [31]. Each mouse was individually placed for six minutes into a 19 cm × 25 cm (diameter × height) plastic cylinder filled with water (at 23–25 °C, 18 cm deep) in dim light. Cylinders were set up in isolated cubicles, and the immobility time was evaluated automatically with the SuperMaze software 3.0. After the test, mice were dried with towels and returned to their individual home cages. The percentage of immobility time during the last five minutes was calculated. An increase in the immobility time indicated depression-like behaviors.

#### 2.4.6. Tail Suspension Test (TST)

The TST was carried out as in the previous report [31]. Mice were suspended on a metal hook in dim light by adhesive tape (1 cm wide) placed 1 cm from the tip of the tail for 6 min. The immobility time was automatically evaluated using the SuperMaze software 3.0. Increased immobility time was considered as symptoms of depression-like behaviors.

#### 2.4.7. Nest Building Test

The nest building test was performed based on previous studies with modification [32,33]. Mice were housed in a clean cage with 12 pieces (6 cm × 6 cm) of paper towel. Nests were evaluated 24 h later on the basis of a 5-point scale: (1) no noticeable removal of paper towels; (2) paper towels scattered throughout the cage; (3) paper towels slightly torn and moved to one corner of the cage; (4) paper towels partially shredded but without any identifiable nest; (5) paper towels mostly shredded into small pieces and arranged into a circular nest. The rater was blinded to the mice groups to eliminate any biases in scoring.

### 2.5. Enzyme-Linked Immunosorbent Assay (ELISA)

The concentrations of TNF-α, IL-1β, IL-6, and IL-10 in peripheral blood samples were measured with respective ELISA kits (Ruixinbio, Quanzhou, China). All the ELISA procedures followed the manufacturer’s instructions.

### 2.6. RNA Extraction and Quantitative Real-Time PCR (qPCR)

After cardiac perfusion with 0.01 M PBS, the brains were harvested, placed into precooled PBS, and sliced into coronal sections (1 mm in thickness) in a mouse brain slice matrix. The prefrontal cortex (PFC), dorsal, and ventral hippocampus were dissected, respectively, and flash frozen in liquid nitrogen and immediately stored at −80 °C until the assay. Total RNA was extracted with TriZol reagent (340405; Life Technologies, Carlsbad, CA, USA), and the reverse transcription was synthesized with EvoScript Universal cDNA Master (07912455001, Roche, Basel, Switzerland) according to standard protocols. The cDNAs of IL-1β, IL-6, TNF-α, and IL-10 were amplified by real-time quantitative PCR (RT-qPCR) using FastStart Universal SYBR Green Master (04913914001, Roche, Basel, Switzerland). The mouse Actin was used as an internal control. The relative gene expression was calculated by the 2^−ΔΔCT^ method. The primers used in RT-qPCR are shown in Table 1.

### 2.7. Western-Blot Analysis

The levels of proteins were analyzed by standard western-blot assays as previously described [34]. In brief, the brain tissue extracts were prepared from −80 °C-stored brain tissue samples with cold RIPA buffer (9806, CST, Danvers, MA, USA) containing protease and phosphatase inhibitors (HY-K0010, HY-K0022; MCE, Monmouth Junction, NJ, USA) and PMSF (8553, CST, Danvers, MA, USA), and rested on ice for 30 min before centrifuging at 12,000× *g* for 20 min at 4 °C. The supernatant was collected, and protein levels were determined with the Enhanced BCA Protein Assay Kit (P0010, Beyotime, Shanghai, China) following the manufacturer’s instructions. Each sample containing 30 μg of protein extract was subjected to SDS-PAGE, using 4–12% Bis-Tris SurePAGE gels (M00652, GenScript, Piscataway, NJ, USA). Then the protein was blotted onto a 0.45 μm PVDF membrane (IPVH00010, Immobilon-P, Millipore, Cork, Ireland) in a cold transfer buffer. Membranes were blocked in 5% bovine serum albumin (BSA; 0332, VWR, Radnor, PA, USA) at room temperature for 60 min and incubated overnight in a specific primary antibody (Iba1, 1:1000, ab5076; Actin, 1:2000, ab8226; Abcam, Cambridge, UK) at 4 °C. Subsequently, the membranes were rinsed in TBST three times (10 min each) and reincubated in a secondary antibody (1:5000; ab6885, ab 6728, Abcam, Cambridge, UK) at room temperature for 60 min. Subsequently, the membranes were rinsed in TBST three times (10 min each) and detected with the enhanced chemiluminescence reagent (ECL) (K-12045-D50, Advansta, Menlo Park, CA, USA). Grayscale analysis was conducted using the Image J (Image J Software, National Institutes of Health, Bethesda, MD, USA)..

### 2.8. Immunofluorescence (IF)

Mouse brain sections were prepared for immunofluorescence. After the mice were completely anesthetized, transcardiac perfusion with 0.01 M PBS was followed by a fixation with 4% paraformaldehyde (PFA) at 4 °C. Brains were quickly removed and fixed in 4% PFA overnight before dehydration in a graded concentration (10–20–30% (*w*/*v*)) of sucrose in 0.1 M PBS (with 0.5% ProClin 300 (48914-U, Sigma-Aldrich, St. Louis, MO, USA)) at 4 °C. The sucrose concentration was replaced when the brains sunk to the bottom. Afterwards, the brains were embedded in O.C.T. compound (Sakura Finetek Japan Co., Ltd., Tokyo, Japan) and sliced into sections of 40 μm in thickness with a Leica CM 1950 microtome (Leica, Wetzlar, Germany). After washes with 0.01 M PBS, the brain sections were blocked in 0.01 M PBS containing 10% donkey serum (SL050, Solarbio, Beijing, China) and 1% BSA plus 0.3% Triton X-100 at room temperature for 1 h. The primary antibody (Iba1, 1:500; 019-19741, Wako, Osaka, Japan) and secondary antibody (Alexa Fluor 594-conjugated anti-rabbit IgG (1:500; A-21207, Invitrogen, Carlsbad, CA, USA)) were both diluted in 0.01 M PBS containing 2.5% donkey serum and 1% BSA plus 0.3% Triton X-100 and, respectively, incubated in a shaker at 4 °C overnight and at room temperature for 1 h. The brain sections were exposed to DAPI (1 μg/mL; 4083, CST, Danvers, MA, USA) for nuclear staining for 5 min, and then cover slips were mounted on slides using an antifade mounting medium (S36963, Invitrogen, Carlsbad, CA, USA). Images of brain slides were obtained using a research slide scanner (VS200, Olympus, Tokyo, Japan), and the number of Iba1-postitve microglia was calculated using Image J.

### 2.9. Assays of Serum Biochemical Parameters

The measurement of aspartate aminotransferase (AST), serum alanine aminotransferase (ALT), Alkaline Phosphatase (AKP/ALP), creatinine, blood urea nitrogen, and uric Acid was performed according to the user guide of the detection kits, respectively (Ruixinbio, Quanzhou, China).

### 2.10. Statistical Analysis

Data were expressed as mean ± SEM and analyzed using GraphPad Prism 9 (GraphPad Software, CA, USA, www.graphpad.com).. Differences between groups and statistical significance were assessed by two-way analysis of variance (ANOVA), with the Bonferroni correction test as the multiple-comparisons post hoc test, by one-way analysis of variance (ANOVA), with the Kruskal–Wallis test as the multiple-comparisons post hoc test, and by unpaired t test. Data beyond the range of ± 2-fold SD were considered as an abnormality and excluded from analyses. A value of *p* < 0.05 was considered statistically significant.

## 3. Results

### 3.1. Preemptive Treatment with GLTs Reduces the Susceptibility to Anxiety-like Behaviors in MS Mice

**Figure 1:**
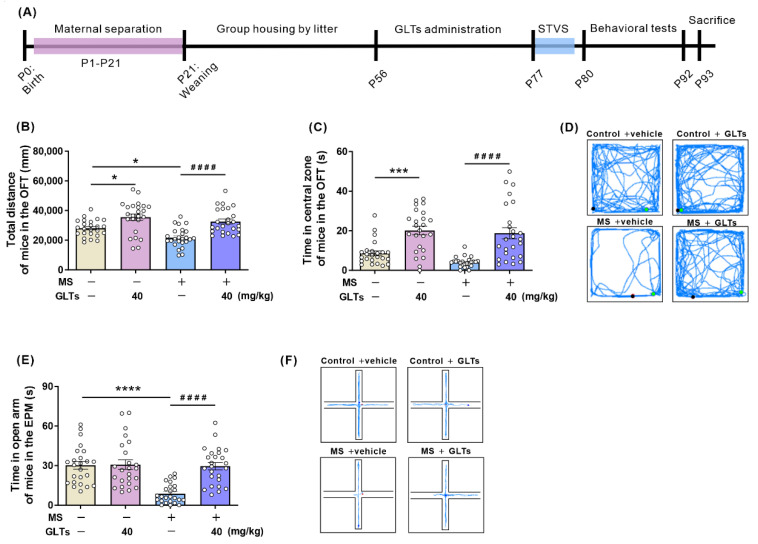
The reduced susceptibility to anxiety-like behaviors in maternally separated (MS) mice by preemptive ganoderma lucidum triterpenoids (GLTs) treatment. (**A**) Schematic diagram of maternal separation, GLTs treatment, sub-threshold variable stress (STVS), and behavioral tests. (**B**) Total distance and (**C**) time spent in the central zone of the open field test (OFT). (**D**) Representative road maps showing the movement trajectory of mice in the OFT. (**E**) Time spent in open arm of the elevated plus maze (EPM). (**F**) The typical movement tracking of mice in the EPM. Data are expressed as mean ± SEM. Control + vehicle group, *n* = 24 (male: *n* = 11; female: *n* = 13); Control + GLTs (40 mg/kg) group, *n* = 24 (male: *n* = 11; female: *n* = 13); MS + vehicle group, *n* = 25 (male: *n* = 12; female: *n* = 13); MS + GLTs (40 mg/kg) group, *n* = 25 (male: *n* = 12; female: *n* = 13). * *p* < 0.05, *** *p* < 0.001, **** *p* < 0.0001, as compared with Control + vehicle group; #### *p* < 0.0001, as compared with MS + vehicle group.

The experimental paradigm is presented in Figure 1A. The evaluation of anxiety-like behaviors in mice was initiated by the OFT at PDN 80 and followed by the EPM two days later, which are two reliable behavioral tests of anxiety. In the open field experiment, no significant interaction between maternal separation and GLTs treatment was observed in all groups when the total distance of spontaneous movements and the time spent in the central zones were analyzed. Further analyses revealed no main effects of maternal separation but significant main effects for GLTs treatment (40 mg/kg) on the spontaneous movements of mice and the time spent in the central zone in the OFT (F (1, 94) = 31.27, *p* < 0.0001; F (1, 94) = 46.43, *p* < 0.0001, respectively) (Figure 1B,C); post-hoc analysis showed that the total travel distance of MS mice markedly decreased when compared with that of the control mice (*p* = 0.0454) and that, after the administration of 40 mg/kg GLTs, the MS mice and control animals both significantly increased their spontaneous movements (*p* < 0.0001; *p* = 0.0114, respectively) and time spent in the central zones (*p* < 0.0001; *p* = 0.0004, respectively) as compared with their counterparts receiving no GLTs treatment. The representative road maps of the movement trajectory in the OFT are depicted in Figure 1D, showing the distinct extensive movement in the OFT in the MS mice receiving the GLTs treatment and the dominant around-edge movement and scarce visits to the central area in those counterparts without GLTs administration. These results suggest that GLTs administration (40 mg/kg) significantly improves the motor capacity and positive mood of the MS and control mice.

Behavioral changes of all the groups in the EPM test are presented in Figure 1E. Maternal separation and GLTs treatment reported a significant interaction (F (1, 94) = 13.05, *p* = 0.0005) and respective main effects on the mice in the EPM test (F (1, 94) = 16.48, *p* = 0.0001; F (1, 94) = 14.54, *p* = 0.0002). The post-hoc analysis showed that MS mice spent less time in the open arms when compared with the control group (*p* < 0.0001) and that the 3-week treatment of 40 mg/kg GLTs significantly increased the time spent in the open arms in the MS mice as compared with their MS counterparts that received no drug administration (*p* < 0.0001). The typical movement tracking of mice is shown in Figure 1F. MS mice rarely entered the open arm and mostly moved or stayed in one place of the closed arm. After the GLTs treatment, the mice tended to explore the open arm more often and farther from the center.

To evaluate the effects of maternal separation and GLTs on the sex of the mice further, we analyzed the behavioral performance of the male and female mice separately. Both male and female MS mice exhibited similar anxiety-like behaviors, which were nearly eliminated by GLTs administration in the OFT (Figure S1A–D) and in the EPM (Figure S1E–F). In our experiments, a GLTs treatment of 40mg/kg showed a better anti-anxiety effect, and lower doses were further investigated for a similar protective effect. The GLTs administration of 20 mg/kg also increased the overall travel distance (*p* = 0.0526) (Figure S2A) and the time spent in the central area (*p* < 0.0001) in the OFT (Figure S2B) and the duration spent in the open arm (*p* < 0.0001) during the EPM (Figure S2C), while a 10 mg/kg GLTs administration did not change any behavior of mice in the OFT and in the EPM. These results indicate that a dose of 40mg/kg of GLTs may produce favorable ameliorating effects on anxiety-like behaviors.

### 3.2. Preemptive Treatment with GLTs Rescues Depression-like Behaviors in MS Mice

Depression-like behaviors were assessed by the SPT, FST, TST, splash test, and nest building following the OFT and EPM.

**Figure 2:**
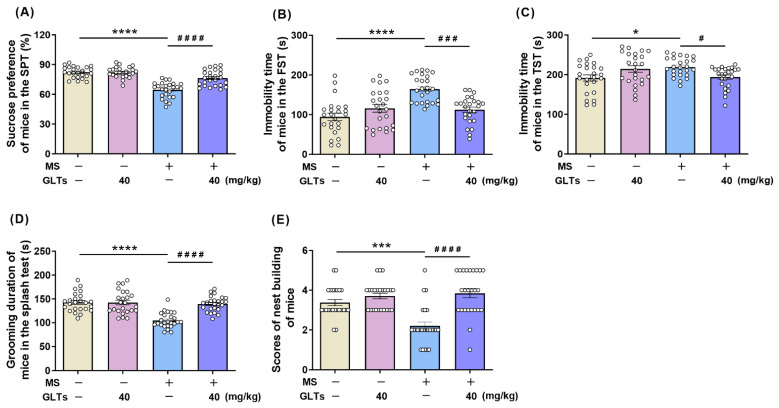
The reduced susceptibility to depression-like behaviors in MS mice by preemptive GLTs treatment. (**A**) Sucrose preference of mice in the SPT. (**B**) Immobility time of mice in the FST. (**C**) Immobility time of mice in the TST. (**D**) Grooming duration of mice in the splash test. (**E**) Nest-building scores of mice in the nest-building test. Data are expressed as mean ± SEM. Control + vehicle group, *n* = 24 (male: *n* = 11; female: *n* = 13); Control + GLTs (40 mg/kg) group, *n* = 24 (male: *n* = 11; female: *n* = 13); MS + vehicle group, *n* = 25 (male: *n* = 12; female: *n* = 13); MS + GLTs (40 mg/kg) group, *n* = 25 (male: *n* = 12; female: *n* = 13). * *p* < 0.05, *** *p* < 0.001, **** *p* < 0.0001, as compared with Control + vehicle group; # *p* < 0.05, ### *p* < 0.001, #### *p* < 0.0001, as compared with MS + vehicle group.

In the SPT, maternal separation and GLTs treatment showed a significant interaction (F (1, 94) = 18.66, *p* < 0.0001) and both had a main effect on the sucrose preference of mice (F (1, 94) = 73.47, F (1, 94) = 18.32, respectively; *p* < 0.0001 for both) (Figure 2A). Despite the adulthood sub-stress, maternal separation in early life induced significantly lower sucrose consumption in the MS mice than in the control animals (*p* < 0.0001), which was markedly improved after the 40 mg/kg GLTs treatment (*p* < 0.0001) (Figure 2A). In the FST, an interaction was evident between maternal separation and GLTs treatment (F (1, 94) = 20.3, *p* = 0.0001), and a main effect was found for maternal separation (F (1, 94) = 16.57, *p* < 0.0001) (Figure 2B). MS mice spent longer immobility time in the FST than control mice (*p* < 0.0001), which was noticeably reduced by the 3-week GLTs treatment (40 mg/kg) (*p* < 0.0001) (Figure 2B). In the TST, maternal separation and GLTs treatment reported no main effect but a significant interaction (F (1, 94) = 13.06, *p* = 0.0005) in which MS mice showed longer immobility time than the control mice (*p* = 0.0255), and the behavior was reduced by the 3-week GLTs treatment (*p* = 0.0477) (Figure 2C).

Furthermore, in the splash test (Figure 2D), a significant interaction was found between maternal separation and GLTs treatment (F (1, 94) = 20.34, *p* < 0.0001). The MS mice showed decreased self-grooming behaviors (*p* < 0.0001), reflecting symptoms of depression such as apathetic behavior. After the 3-week 40 mg/kg GLTs treatment, the self-grooming behaviors increased, and the apathetic behavior was alleviated (*p* < 0.0001). Nest building behaviors can be regarded as a measure of the well-being of the mice, and impaired nest building can be taken to represent a negative phenotype of psychiatric diseases including depression. In the nest building test (Figure 2E), an interaction was evident between maternal separation and GLTs treatment in terms of the self-care behaviors of mice (F (1, 96) = 12.61, *p* = 0.0006), in which the MS mice had significantly lower nesting scores than the control mice (*p* = 0.0001), while the preemptive GLTs treatment (40 mg/kg) greatly improved the nesting scores (*p* < 0.0001). Taken together, these findings strongly indicate that GLTs treatment can markedly ameliorate the MS-induced depression-like behaviors in the mice.

Similar to the assessment of anxiety-like behaviors, male and female MS mice displayed similar behaviors in the SPT, FST, TST, splash test, and nest building test. Despite no statistical difference in the immobility time in the TST (Figure S3A,B), both male and female MS animals exhibited decreased sucrose consumption in the SPT (Figure S3C,D), increased immobility time in the FST (Figure S3E,F), less grooming duration in the splash test (Figure S3G,H), and lower nest scores in the nest building test (Figure S3I,J), which were all rescued by the 3-week 40 mg/kg GLTs treatment. The 20 mg/kg GLTs administration exerted no effects on the sucrose consumption (Figure S4A) but certain beneficial effects on the depression-like behaviors of the MS mice in the FST, TST, splash test, and nest building test. The 20 mg/kg GLTs administration decreased the immobility time in the FST (*p* = 0.0009) (Figure S4B) and TST (*p* = 0.0008) (Figure S4C), and improved the grooming duration in the splash test (*p* < 0.0001) (Figure S4D) and the nesting scores in the nest building test (*p* = 0.0077) (Figure S4E). The 10 mg/kg GLTs administration had a partial effect on the immobility time in the TST (*p* = 0.0164) (Figure S4C) and grooming duration in the splash test (*p* = 0.0031) (Figure S4D).

Altogether, the findings from all the behavioral tests above suggest that, regardless of sex differences, GLTs can alleviate the anxiety- and depression-like behaviors of mice that are subjected to maternal separation with the 40 mg/kg of GLTs boasting the most favorable effects.

### 3.3. GLTs Reduces the Peripheral Inflammatory Response in MS Mice

**Figure 3:**
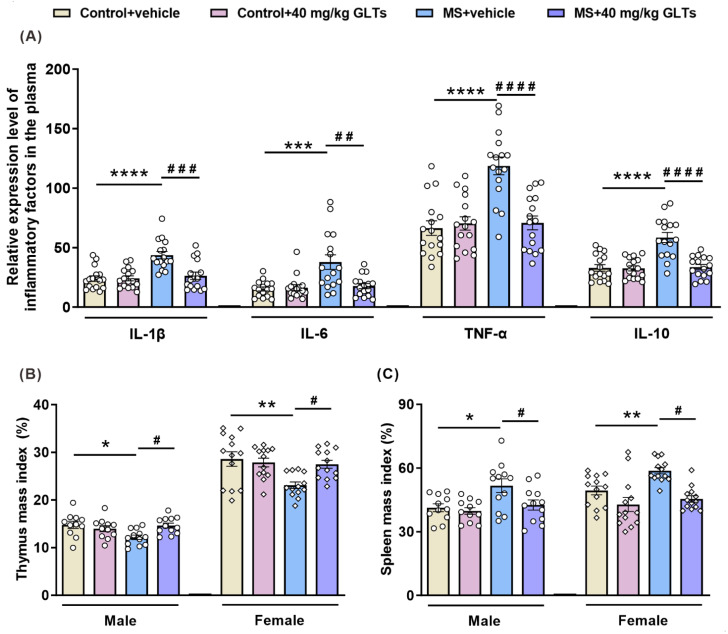
The reduced peripheral inflammatory response in MS mice by GLTs treatment. (**A**) IL-1β, IL-6, TNF-α, and IL-10 level in the periphery of mice. Data are expressed as mean ± SEM, *n* = 16/group. (**B**) The thymus mass index of the male and female mice. (**C**) The spleen mass index of the male and female mice. Data are expressed as mean ± SEM. Male: Control + vehicle group, *n* = 11; Control + GLTs (40 mg/kg) group, *n* = 11; MS + vehicle group, *n* = 12; MS + GLTs (40 mg/kg) group, *n* = 12. Female: *n* = 13/group. * *p* < 0.05, ** *p* < 0.01, *** *p* < 0.001, **** *p* < 0.0001, as compared with Control + vehicle group; # *p* < 0.05, ## *p* < 0.01, ### *p* < 0.001, #### *p* < 0.0001, as compared with MS + vehicle group.

To explore the role of maternal separation and GLTs treatment in anxiety- and depression-like behaviors of mice, the serum levels of the inflammatory factors IL-1β, IL-6, TNFα, and IL-10 were measured by ELISA (Figure 3A). The analysis revealed a significant interaction between maternal separation and GLTs treatment (IL-1β: F (1, 60) = 10.76, *p* = 0.0017; IL-6: F (1, 60) = 8.977, *p* = 0.0040; TNFα: F (1, 60) = 17.17, *p* = 0.0001; IL-10: F (1, 60) = 16.93, *p* = 0.0001). The expression of IL-1β (*p* < 0.0001), IL-6 (*p* = 0.0002) and TNFα (*p* < 0.0001) significantly increased in the serum of MS mice, indicating an augmented inflammatory response. After the GLTs administration, the expression of these inflammatory factors gradually returned to a normal level (IL-1β: *p* = 0.0002; IL-6: *p* = 0.0010; TNFα: *p* < 0.0001). Of interest, as a typical anti-inflammatory factor, the level of IL-10 was also significantly higher in the MS mice than in the control group (*p* < 0.0001), which was reversed by the GLTs treatment (*p* < 0.0001). These findings suggest that maternal separation may induce parallelly promoted pro-inflammatory and anti-inflammatory responses in MS mice, which can be downregulated by GLTs treatment, resulting in no excess inflammatory responses in vivo.

As the thymus and spleen are two important immune organs, maternal separation and GLTs treatments may affect the indexes of the thymus and spleen, which reflect the damage or recovery of the immune system. The results showed that the functions of the thymus and spleen were inhibited after maternal separation. An interaction between maternal separation and GLTs treatment was found in thymic indexes of the male and female mice (male: F (1, 42) = 7.591, *p* = 0.0086; female: F (1, 48) = 6.113, *p* = 0.0170) with a dramatic thymic atrophy observed in mice that suffered from MS without GLTs treatment (male: *p* = 0.0198; female: *p* = 0.0028) (Figure 3B). An interaction between maternal separation and GLTs treatment was also reported in male spleen indexes (F (1, 42) = 4.415, *p* = 0.0417), and both maternal separation and GLTs treatment had a main effect on the spleen indexes (Figure 3C). After maternal separation, the spleen underwent a compensatory hypertrophy (male: *p* = 0.0222; female: *p* = 0.0263). After the 3-week 40 mg/kg GLTs treatment, the indexes of the thymus and spleen were significantly reversed and normalized (thymus: male, *p* = 0.0270; female, *p* = 0.0257; spleen: male, *p* = 0.0069; female, *p* = 0.0093). These results suggest that GLTs treatment may restore the function of the thymus and spleen in MS mice.

### 3.4. GLTs Balances the Expression of Pro-Inflammatory and Anti-Inflammatory Factors in the PFC and Hippocampus of MS Mice

We also examined the mRNA level of inflammation-related factors, including IL-1β, IL-6, TNFα, and IL-10 in the PFC, dorsal hippocampus, and ventral hippocampus, which are three critical regions of the brain that are related to anxiety and depression.

**Figure 4:**
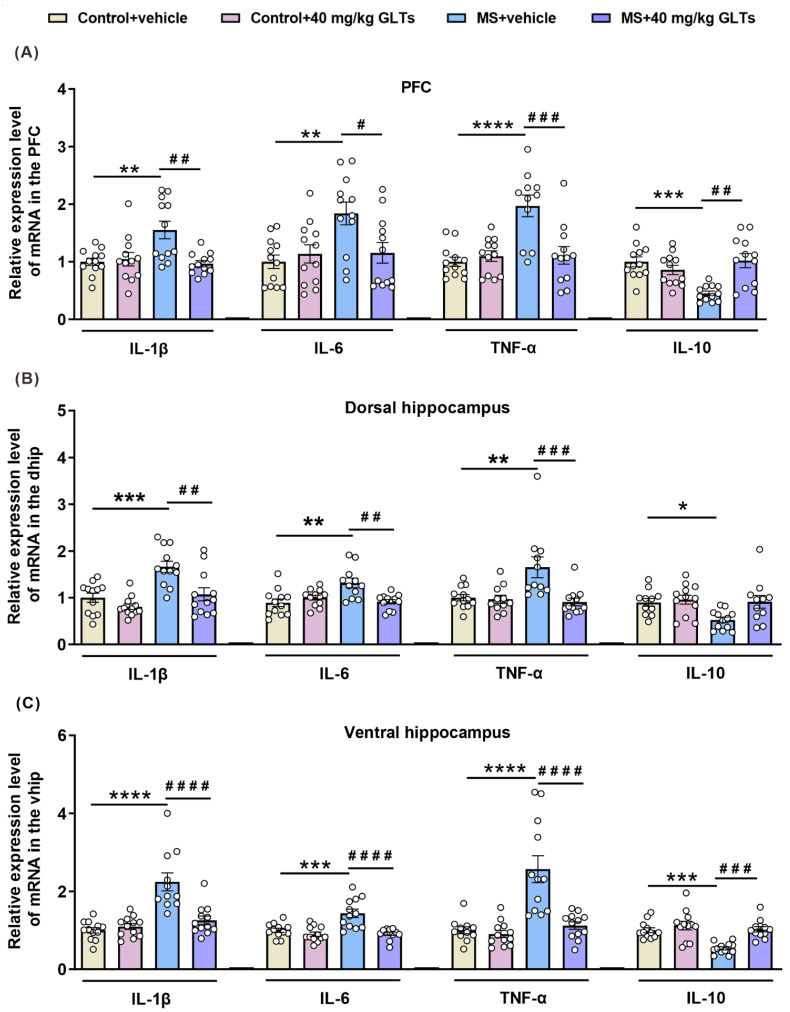
The GLTs-balanced expressions of inflammatory and anti-inflammatory factors in the PFC and hippocampus of the MS mice. (**A**–**C**) IL-1β, IL-6, TNF-α, and IL-10 level in the PFC (**A**), dorsal hippocampus (**B**), and ventral hippocampus (**C**) of mice. Data are expressed as mean ± SEM, *n* = 12/group. * *p* < 0.05, ** *p* < 0.01, *** *p* < 0.001, **** *p* < 0.0001, as compared with Control + vehicle group; # *p* < 0.05, ## *p* < 0.01, ### *p* < 0.001, #### *p* < 0.0001, as compared with MS + vehicle group.

In the PFC of mice (Figure 4A), an interaction between maternal separation and GLTs treatment was reported in the mRNA levels of IL-1β (F (1, 44) = 9.025, *p* =0.0044), IL-6 (F (1, 44) = 6.122, *p* = 0.0173), TNF-α (F (1, 43) = 13.08, *p* = 0.0008), and IL-10 (F (1, 44) = 16.68, *p* = 0.0020). Maternal separation had a main effect on the levels of IL-1β (F (1, 44) = 5.075, *p* = 0.0293), IL-6 (F (1, 44) = 6.677, *p* = 0.0132), TNF-α (F (1, 43) = 13.77, *p* = 0.0006), and IL-10 (F (1, 44) = 4.984, *p* = 0.0307). Similarly, the GLTs treatment also exerted a main effect on the expression of IL-1β (F (1, 44) = 6.644, *p* = 0.0134), TNF-α (F (1, 43) = 8.274, *p* = 0.0062), and IL-10 (F (1, 44) = 6.012, *p* = 0.0182). The MS mice showed an increased expression of inflammatory markers IL1β (*p* = 0.0031), IL-6 (*p* = 0.0052), and TNF-α (*p* < 0.0001) and a decreased expression of anti-inflammatory marker IL-10 (*p* = 0.0003). After the 3-week 40 mg/kg GLTs treatment, the levels of inflammatory markers decreased (IL-1β: *p* = 0.0016; IL-6: *p* = 0.0330; TNF-α: *p* = 0.0003), and the level of anti-inflammatory IL-10 increased (*p* = 0.0002).

In the dorsal hippocampus of mice (Figure 4B), an interactive effect between maternal separation and GLTs treatment was found on the mRNA level of IL-6 (F (1, 43) = 12.34, *p* = 0.0016) and TNF-α (F (1, 42) = 7.819, *p* = 0.0078). Maternal separation had main effects on the mRNA expression of IL-1β (F (1, 44) = 18.69, *p* < 0.0001), IL-6 (F (1, 43) = 5.334, *p* = 0.0187), TNF-α (F (1, 42) = 5.46, *p* = 0.0242), and IL-10 (F (1, 43) = 5.833, *p* = 0.0200), and GLTs treatment had main effects on the mRNA level of IL-1β (F (1, 44) = 13.37, *p* = 0.0007) and TNF-α (F (1, 42) = 9.346, *p* = 0.0338). In MS mice, increased expressions of the cytokines IL-1β, IL-6, and TNF-α were observed (IL-1β: *p* = 0.0004; IL-6: *p* = 0.0012; TNF-α: *p* = 0.0046, IL-10: *p* = 0.0175) while the level of IL-10 decreased (IL-10: *p* = 0.0175); the GLTs treatment did not affect the expression of IL-10 but reduced the increased expressions of the other cytokines (IL-1β: *p* = 0.0018; IL-6: *p* = 0.0024; TNF-α: *p* = 0.0010).

In the ventral hippocampus of mice (Figure 4C), a significant interaction between maternal separation and GLTs treatment was found on the level of the inflammatory factors (IL-1β: F (1, 44) = 16.02, *p* = 0.0002; IL-6: F (1, 44) = 9.368, *p* = 0.0038; TNF-α: F (1, 44) =12.32, *p* = 0.0011; IL-10: F (1, 44) = 5.917, *p* = 0.0191); both maternal separation and GLTs treatment, respectively, had main effects on the mRNA expressions of IL-1β (F (1, 44) = 25.1, *p* < 0.0001; F (1, 44) = 11.52, *p* = 0.0015), IL-6 (F (1, 44) = 10.51, *p* = 0.0023; F (1, 44) = 20.32, *p* < 0.0001), TNF-α (F (1, 44) =21.43, *p* < 0.0001; F (1, 44) = 16.01, *p* = 0.0002), and IL-10 (F (1, 44) = 13.56, *p* = 0.0006; F (1, 44) = 16.43, *p* = 0.0002). Compared with that of the control group, the ventral hippocampus of the MS mice exhibited a significant increase in the mRNA level of IL-1β (*p* < 0.0001), IL-6 (*p* = 0.0003), TNFα (*p* < 0.0001), and a decrease in the expression of IL-10 (*p* = 0.0005), indicating an augmented inflammatory response. After the 3-week GLTs treatment, the mRNA levels of all the markers were normalized (IL-1β: *p* < 0.0001; IL-6: *p* < 0.0001; TNF-α: *p* < 0.0001; IL-10: *p* = 0.0002).

These data suggest that a wide range of changes in the mRNA level of pro-inflammatory and anti-inflammatory markers may occur in the MS mice, which may be counterbalanced by GLTs treatment.

### 3.5. GLTs Inhibit the Maternal Separation-Induced Activation of the Microglia in the PFC and Hippocampus

**Figure 5:**
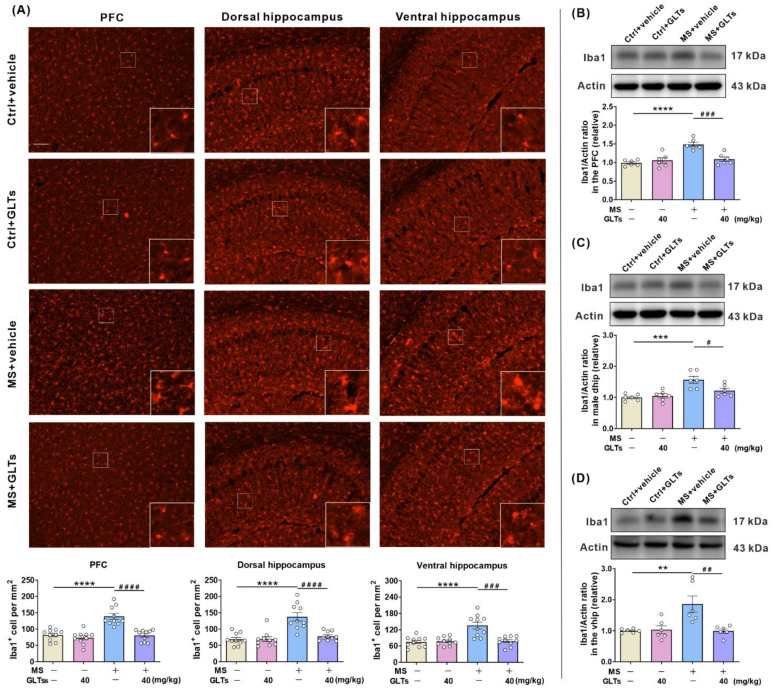
The inhibited maternal separation-induced microglial activation in the PFC and hippocampus by GLTs treatment. (**A**) Representative images of microglia and quantification analysis of microglia number in the PFC, dorsal, and ventral hippocampus of MS and control mice. Scale bar, 100 μm. (**B**–**D**) Representative western-blot and quantification of Iba1 in the PFC (**B**), dorsal hippocampus (**C**), and ventral hippocampus (**D**). Data are expressed as mean ± SEM, *n* = 10/group. ** *p* < 0.01, *** *p* < 0.001, **** *p* < 0.0001, as compared with Control + vehicle group; # *p* < 0.05, ## *p* < 0.01, ### *p* < 0.001, #### *p* < 0.0001, as compared with MS + vehicle group.

It is known that microglia are the main modulator of inflammation in the brain, and the activation of microglia is also associated with enhanced inflammatory reactions. Immunofluorescence staining of Iba1 was performed to evaluate the number and activation of microglia in the PFC and hippocampus, and Iba1-positive microglia were quantified. After maternal separation, the microglia in the PFC and hippocampus displayed larger cell bodies and thicker processes, consistent with an activated morphological profile; after the 3-week GLTs treatment, the morphology of the microglia returned to the resting configuration (Figure 5A). The statistical analysis of the number of microglia was subsequently carried out. The data showed an interactive effect between maternal separation and GLTs treatment on the number of the microglia in the PFC (F (1, 36) = 13.26, *p* = 0.0008), dorsal hippocampus (F (1, 36) = 13.84, *p* = 0.0007), and ventral hippocampus (F (1, 36) =13.45, *p* = 0.0008) of the mice. Both maternal separation and GLTs treatment had respective main effects on the number of Iba1-positive microglia in the PFC (F (1, 36) = 21.29, *p* < 0.0001; F (1, 36) = 23.99, *p* < 0.0001), dorsal hippocampus (F (1, 36) = 23.44, *p* < 0.0001; F (1, 36) = 13.56, *p* = 0.0008), and ventral hippocampus (F (1, 36) = 14.1, *p* = 0.0006; F (1, 36) = 11.22, *p* = 0.0019). The post-hoc analysis reported a significant increase in the number of Iba1-positive microglia (*p* < 0.0001). After the 3-week GLTs treatment, the number of Iba1-positive microglia in the PFC (*p* < 0.0001), dorsal hippocampus (*p* < 0.0001), and ventral hippocampus (*p* = 0.0001) gradually returned to a normal level.

The protein level of Iba1 in the three brain regions was detected. Compared with the mice without maternal separation, the MS mice exhibited a significantly higher level of Iba1 in the PFC (*p* < 0.0001) and dorsal hippocampus (*p* = 0.0004) and ventral hippocampus (p = 0.0035), which was reversed by GLTs (PFC: *p* < 0.0007; dorsal hippocampus: *p* <0.0344; ventral hippocampus: *p* = 0.0032) (Figure 5B–D). These data demonstrate that after maternal separation, the number and protein levels of microglia in the PFC and hippocampus of mice increase, and GLTs treatment can significantly reduce the activation of microglia.

### 3.6. GLTs Have No Adverse Effects on Body Weight and the Functions of Liver and Kidneys

**Figure 6:**
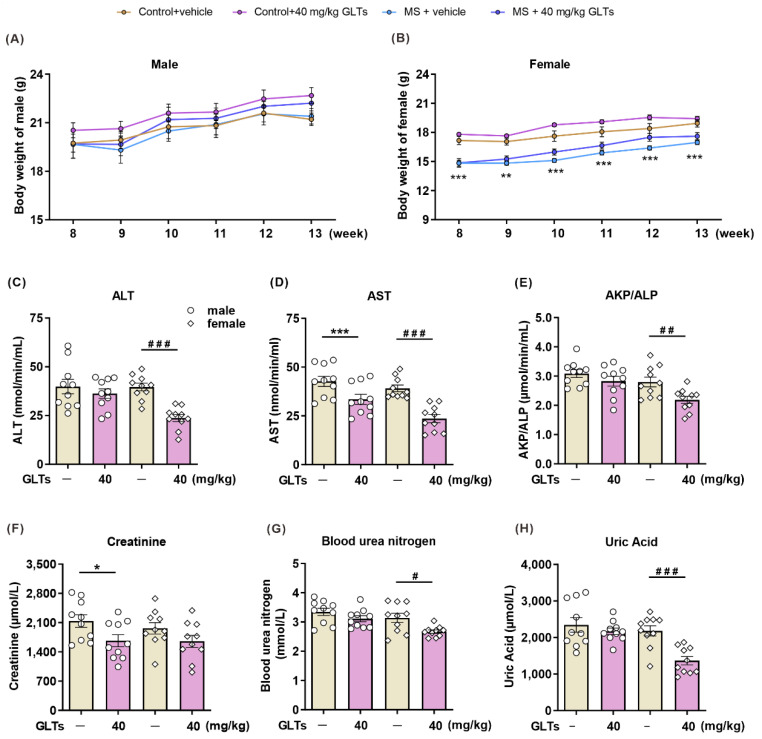
No adverse effects of GLTs on body weight and the functions of liver and kidneys. (**A**,**B**) Body weight of male (**A**) and female (**B**) mice from 8th week to 13th week. Data are expressed as mean ± SEM, *n* = 11-13/group. ** *p* < 0.01 *** *p* < 0.001, as compared with female Control + vehicle group. (**C**–**E**) The level of liver damage biomarkers ALT (**C**), AST (**D**), and AKP/ALP (**E**) in the plasma of male and female mice. (**F**–**H**) The level of kidney damage biomarkers creatinine (**F**), blood urea nitrogen (**G**), and uric acid (**H**) in plasma of male and female mice. Data are expressed as mean ± SEM, *n* = 10/group. * *p* < 0.05, *** *p* < 0.001, as compared with male Control + vehicle group; # *p* < 0.05, ## *p* < 0.01, ### *p* < 0.001, as compared with female Control + vehicle group.

The changes in body weight in all groups were monitored during the treatment period (Figure 6A,B). Compared with their control groups, male and female MS mice displayed a significant decrease in body weight throughout childhood and adolescence (data not shown). During adulthood, no significant changes in body weight were observed in male mice, regardless of either maternal separation or drug administration (Figure 6A), while the female MS mice featured a significantly lower body weight when compared with the control group, though they showed an insignificant increase after the drug treatments (Figure 6B). These results suggest that GLTs treatment exerts no significant effect on the body weight of the MS mice.

We also examined changes in liver and kidney function in mice after the treatment with GLTs (Figure 6C–H). Hepatocellular injury often results in an increase in serum ALT and AST, so plasma ALT and AST levels are the most important and sensitive liver damage biomarkers. AKP/ALP is ubiquitously expressed in all tissues and mainly in the liver. The level of AKP/ALP in plasma is elevated when hepatobiliary disease occurs. After the 3-week GLTs administration, the activities of AST, ALT, and AKP/ALP in the plasma of female mice decreased when compared with those of the control groups (*p* < 0.0001; *p* < 0.0001; *p* = 0.0085, respectively) (Figure 6C–E). The plasma level of AST in male mice also showed a lower activity than in the control group (*p* = 0.0113). Serum creatinine, blood urea nitrogen, and uric acid, which are important markers of renal function, tend to increase when abnormalities occur in the kidneys. After the 3-week administration of GLTs, the level of creatinine in the male mice was significantly reduced (*p* = 0.0363) (Figure 6F), and the levels of blood urea nitrogen (*p* = 0.0115) (Figure 6G) and uric acid (*p* = 0.0003) (Figure 6H) were decreased in the female mice. These damaging markers of the liver and kidneys remained at a lower level after the GLTs treatment, indicating that GLTs not only produce no toxic effects on the liver and kidneys of the mice, but also preserve their functions to a certain extent.

## 4. Discussion

Early life experiences can impact brain function and behavior [35,36,37]. In the human populace, stressful early life events, including maternal negligence or childhood abuse, may increase the risk of adulthood mood disorders [2,38]. Our results showed that when exposed to the adulthood sub-stress, the MS male and female mice, compared with the control groups, displayed anxiety- and depression-like behaviors in the various behavioral tests and reported significantly elevated pro-inflammatory cytokines in the periphery and the cerebral regions of the PFC and hippocampus, in which an association was evident between the microglial activation and the abnormal behavioral performances. These peripheral and cerebral inflammatory processes were inhibited by GLTs administration, and the maternal-separation-induced behavioral abnormalities were reversed.

As the most commonly employed pre-clinical approach to the neuropsychiatric consequences of early-life stress [39,40,41], maternal separation in early life can subject the offspring to long-term mental disorders such as depression and anxiety [28,40,42,43,44]. The duration and number of days of the separation period are critical factors for maternal-separation-related anxiety and depression in individuals, with longer separation periods leading to severer emotional disorders [45]. In this study, we chose a longer separation period, that is, 4 h per day from PND 1–21, which is consistent with the real-life phenomenon that many children are unable to change their living environment before adulthood. Female subjects have been found to be more prone to stress-related psychiatric disorders at a female/male risk ratio of roughly 2:1 [46,47]. Recent studies of rodents evidenced sex differences in neurobiological susceptibility to ELS [27,48,49,50]. Thus, in this study, we differentiated male and female mice to evaluate differences in anxiety- and depression-like behaviors and biological effects following maternal separation and GLTs treatment. However, our results showed that both males and females exhibited similar behavioral abnormalities and biological responses after the stress exposure, which were both similarly reversed by the GLTs treatment. This discrepancy may indicate that maternal separation can indeed induce a strong impact on the adult mice regardless of their sex difference, and GLTs have remarkable preventive and therapeutic effects on both sexes.

A second stress in adulthood subsequent to childhood adversity has been thought to be an important trigger for behavioral abnormalities and psychiatric disorders [51]. In this study, a mouse stress paradigm of “maternal separation in childhood + sub-stress in adulthood” was employed, and several classical indicators were adopted to assess the behaviors of mice, which successfully produced the anxiety- and depressive-like behaviors. Our results are consistent with the notion that the “first hit” of maternal separation may render individuals more susceptible to the negative experience of a “second hit” [52].

In our study, the peripheral inflammatory factors including TNFα, IL-1β, and IL-6 were significantly upregulated in MS mice, which is in line with those of a clinical report that individuals who experienced early life adversity exhibited a marked increase in the peripheral of CRP, IL-6, and TNF-α in comparison with those who did not [53]. Another previous study documented an elevated IL-6 response (higher IL-6 concentration and greater acute IL-6 release) to acute psychosocial stresses in the individuals exposed to moderate-severe childhood maltreatment experiences in comparison with the healthy controls [54]. Of interest, in this study, as a typical anti-inflammatory factor, the level of IL-10 in the MS mice was also significantly upregulated when compared with that of the control group, which is inconsistent with previous reports [55]. We also observed a decrease in the mRNA level in the brain of MS mice. One potential explanation for this inconsistency may lie in the fact we examined the protein of IL-10, instead of its mRNA, in the periphery. Compared with the brain, the peripheral immune system may also respond to the elevated inflammatory factors more vigorously. However, unfortunately, the elevated IL-10 cannot curb the elevated pro-inflammatory factors, and the pro- and anti-inflammatory responses were parallelly promoted in the periphery of MS mice. Alternatively interpreted, the results reveal that it is either in the periphery or in the brain of the MS mice that the balance between pro- and anti-inflammatory responses is perturbed. This difference may be caused by different species, modeling methods, and detection times. Moreover, the current study reported significantly increased mRNA levels of inflammatory factors in the PFC and hippocampus of MS mice, indicating a marked cerebral inflammatory response. Indeed, the available literature documents that microglia are an important source of cytokines and the vital inflammatory mediators in the central nervous system [56,57]. The current study found that microglia proliferated in the PFC and hippocampus of both male and female MS mice. The activation of microglia alters the balance between pro- and anti-inflammatory cytokines in the PFC and hippocampus, favoring the expression of pro-inflammatory cytokines. In the light of these neuroinflammatory processes and behavioral abnormalities in the MS mice, our study suggests that maternal separation in childhood may increase inflammation-mediated susceptibility to stress in adulthood, which echoes the findings of the previous research [50,51,58,59].

*G. lucidum* is considered to be a potent medicinal source due to its abundance of pharmacological active compounds. Triterpenes are one of the main and important active ingredients of *G. lucidum*, which can inhibit inflammatory responses, including peripheral [60] and brain inflammation. Previous studies show that ganoderic acid A ameliorates LPS-induced neuroinflammation by activating the farnesoid x receptor in vitro [61] and balances the Th17/Tregs axis to attenuate the neuroinflammation of Alzheimer’s disease in mice [23]. Deacetyl ganoderic acid F, another kind of triterpene obtained from *G. lucidum*, was found to suppress LPS-induced neural inflammation in vivo and significantly reduce the expression of inflammatory factors in the peripheral serum of mice [25]. A new triterpenoid from *G. lucidum*, named Ganoderterpene A, was evidenced to protect microglia from apoptosis by restraining the inflammatory response [12]. In our study, GLTs were systemically administered, which is consistent with the clinical medication practices of traditional Chinese medicine [10,62]. The preemptive treatment with GLTs decreased the expressions of peripheral inflammatory factors, alleviating the inflammatory responses in MS mice. In the PFC and hippocampus, GLTs treatment exactly rescued the increase in the mRNA levels of inflammatory factors. Furthermore, GLTs pretreatment restored the number and resting state of microglia in the MS mice in comparison with those of the control group, featuring a smaller cell body and higher ramification, which suggests that GLTs can reduce the stress sensitivity and improve the abnormal behaviors in adulthood by inhibiting the activation of microglia in MS mice.

Recent research efforts into GLTs focus more on their pharmacological properties in treating acute or chronic diseases but less on the side effects and toxicity. In the current study, close attention was steered to the effects of GLTs on the body weight and directly related organs of mice. Our study showed that GLTs slightly increased the body weight of mice in all groups and ameliorated the atrophic thymus and compensative hypertrophy of the spleen in the MS mice. The reduction in the expressions of some damage indicators for liver and kidney function, especially in female MS mice, suggests that GLTs may have a certain protective effect on the liver and kidneys.

## 5. Conclusions

In summary, our results suggest that GLTs can mitigate the anxiety- and depression-like behaviors in MS mice by reducing peripheral and cerebral inflammation without evident toxicities on the vital organs. The findings offer novel insights into the clinical value of GLTs as a promising candidate in treating ELS-induced anxiety and depression. At present, the clinical use of GLTs is only prevalent in Asia, especially East Asia. More efforts and research, especially clinical trials, are awaited to confirm the safety and efficacy for global clinical promotion.

## Figures and Tables

**Table 1:** Primers of investigated genes in qPCR analysis.

Gene		Primer Sequences
Actin	Forward	TGTCCACCTTCCAGCAGATGT
	Reverse	AGCTCAGTAACAGTCCGCCTAG
IL-1β	Forward	TCGCAGCAGCACATCAACAAGAG
	Reverse	AGGTCCACGGGAAAGACACAGG
IL-6	Forward	CTCCCAACAGACCTGTCTATAC
	Reverse	CCATTGCACAACTCTTTTCTCA
TNF-α	Forward	ACTGGCAGAAGAGGCACTCC
	Reverse	GCCACAAGCAGGAATGAGAA
IL-10	Forward	GCTCTTACTGACTGGCATGAG
	Reverse	CGCAGCTCTAGGAGCATGTG

## Data Availability

Not applicable.

## Supplementary Materials

The following supporting information can be downloaded at: https://www.mdpi.com/article/10.3390/nu14112268/s1, Figure S1: The similar effects of maternal separation and GLTs treatment on the anxiety-like behaviors of the male and female mice; Figure S2: Effects of different doses of GLTs on anxiety-like behaviors of MS mice; Figure S3: Similar effects of maternal separation and GLTs treatment on the depression-like behaviors in male and female mice; Figure S4: Effects of different doses of GLTs on depression-like behaviors in MS mice; Detailed analysis results of GLTs mentioned in Section 2.1.

## References

[1] Kessler R.C., McLaughlin K.A., Green J.G., Gruber M.J., Sampson N.A., Zaslavsky A.M., Aguilar-Gaxiola S., Al-Hamzawi A.O., Alonso J., Angermeyer M. (2010). Childhood adversities and adult psychopathology in the WHO World Mental Health Surveys. Br. J. Psychiatry.

[2] Li M., Darcy C., Meng X. (2015). Maltreatment in childhood substantially increases the risk of adult depression and anxiety in prospective cohort studies: Systematic review, meta-analysis, and proportional attributable fractions. Psychol. Med..

[3] LeMoult J., Humphreys K.L., Tracy A., Hoffmeister J.-A., Ip E., Gotlib I.H. (2019). Meta-analysis: Exposure to early life stress and risk for depression in childhood and adolescence. J. Am. Acad. Child. Adolesc. Psychiatry.

[4] Mandelli L., Petrelli C., Serretti A. (2015). The role of specific early trauma in adult depression: A meta-analysis of published literature. Childhood trauma and adult depression. Eur. Psychiatry.

[5] Infurna M.R., Reichl C., Parzer P., Schimmenti A., Bifulco A., Kaess M. (2016). Associations between depression and specific childhood experiences of abuse and neglect: A meta-analysis. J. Affect. Disord..

[6] Danese A., Pariante C.M., Caspi A., Taylor A., Poulton R. (2007). Childhood maltreatment predicts adult inflammation in a life-course study. Proc. Natl. Acad. Sci. USA.

[7] Pace T.W., Mletzko T.C., Alagbe O., Musselman D.L., Nemeroff C.B., Miller A.H., Heim C.M. (2006). Increased stress-induced inflammatory responses in male patients with major depression and increased early life stress. Am. J. Psychiatry.

[8] Miller G.E., Chen E. (2010). Harsh family climate in early life presages the emergence of a proinflammatory phenotype in adolescence. Psychol. Sci..

[9] Taylor S.E., Lehman B., Kiefe C.I., Seeman T.E. (2006). Relationship of early life stress and psychological functioning to adult c-reactive protein in the coronary artery risk development in young adults study. Biol. Psychiatry.

[10] Ahmad F. (2018). Ganoderma lucidum: Persuasive biologically active constituents and their health endorsement. Biomed. Pharmacother..

[11] Wu G.-S., Guo J.-J., Bao J.-L., Li X.-W., Chen X.-P., Lu J.-J., Wang Y.-T. (2013). Anti-cancer properties of triterpenoids isolated from *Ganoderma lucidum*—A review. Expert Opin. Investig. Drugs.

[12] Kou R.-W., Gao Y.-Q., Xia B., Wang J.-Y., Liu X.-N., Tang J.-J., Yin X., Gao J.-M. (2021). Ganoderterpene A, a New Triterpenoid from *Ganoderma lucidum*, Attenuates LPS-induced inflammation and apoptosis via suppressing MAPK and TLR-4/NF-κB pathways in BV-2 cells. J. Agric. Food Chem..

[13] Wu Y.-L., Han F., Luan S.-S., Ai R., Zhang P., Li H., Chen L.-X. (2019). Triterpenoids from *Ganoderma lucidum* and their potential anti-inflammatory effects. J. Agric. Food Chem..

[14] Hsu P.-L., Lin Y.-C., Ni H., Mo F.-E. (2018). Ganoderma triterpenoids exert antiatherogenic effects in mice by alleviating disturbed flow-induced oxidative stress and inflammation. Oxidative Med. Cell. Longev..

[15] Guo W.-L., Guo J.-B., Liu B.-Y., Lu J.-Q., Chen M., Liu B., Bai W.-D., Rao P.-F., Ni L., Lv X.-C. (2020). Ganoderic acid A from Ganoderma lucidum ameliorates lipid metabolism and alters gut microbiota composition in hyperlipidemic mice fed a high-fat diet. Food Funct..

[16] Wang C., Liu X., Lian C., Ke J., Liu J. (2019). Triterpenes and aromatic meroterpenoids with antioxidant activity and neuroprotective effects from Ganoderma lucidum. Molecules.

[17] Zhu M., Chang Q., Wong L.K., Chong F.S., Li R.C. (1999). Triterpene antioxidants from ganoderma lucidum. Phytother Res.

[18] Ahmad F., Ahmad F.A., Khan M.I., Alsayegh A.A., Wahab S., Alam M.I., Ahmed F. (2021). Ganoderma lucidum: A potential source to surmount viral infections through β-glucans immunomodulatory and triterpenoids antiviral properties. Int. J. Biol. Macromol..

[19] Zhao C., Fan J., Liu Y., Guo W., Cao H., Xiao J., Wang Y., Liu B. (2018). Hepatoprotective activity of Ganoderma lucidum triterpenoids in alcohol-induced liver injury in mice, an iTRAQ-based proteomic analysis. Food Chem..

[20] Su L., Liu L., Jia Y., Lei L., Liu J., Zhu S., Zhou H., Chen R., Lu H.A.J., Yang B. (2017). Ganoderma triterpenes retard renal cyst development by downregulating Ras/MAPK signaling and promoting cell differentiation. Kidney Int..

[21] Shao G., He J., Meng J., Ma A., Geng X., Zhang S., Qiu Z., Lin D., Li M., Zhou H. (2021). Ganoderic Acids Prevent Renal Ischemia Reperfusion Injury by Inhibiting Inflammation and Apoptosis. Int. J. Mol. Sci..

[22] Yu N., Huang Y., Jiang Y., Zou L., Liu X., Liu S., Chen F., Luo J., Zhu Y. (2020). Ganoderma lucidum Triterpenoids (GLTs) reduce neuronal apoptosis via inhibition of ROCK signal pathway in APP/PS1 transgenic alzheimer’s disease mice. Oxidative Med. Cell. Longev..

[23] Zhang Y., Wang X., Yang X., Yang X., Xue J., Yang Y. (2021). Ganoderic Acid A To Alleviate Neuroinflammation of alzheimer’s disease in mice by regulating the imbalance of the Th17/Tregs axis. J. Agric. Food Chem..

[24] Abulizi A., Ran J., Ye Y., An Y., Zhang Y., Huang Z., Lin S., Zhou H., Lin D., Wang L. (2021). Ganoderic acid improves 5-fluorouracil-induced cognitive dysfunction in mice. Food Funct..

[25] Sheng F., Zhang L., Wang S., Yang L., Li P. (2019). Deacetyl ganoderic acid f inhibits LPS-induced neural inflammation via NF-κB pathway both in vitro and in vivo. Nutrients.

[26] Peña C.J., Smith M., Ramakrishnan A., Cates H.M., Bagot R.C., Kronman H.G., Patel B., Chang A.B., Purushothaman I., Dudley J. (2019). Early life stress alters transcriptomic patterning across reward circuitry in male and female mice. Nat. Commun..

[27] Hodes G., Pfau M.L., Purushothaman I., Ahn H.F., Golden S.A., Christoffel D.J., Magida J., Brancato A., Takahashi A., Flanigan M.E. (2015). Sex differences in nucleus Accumbens transcriptome profiles associated with susceptibility versus resilience to Subchronic variable stress. J. Neurosci..

[28] Peña C.J., Kronman H.G., Walker D.M., Cates H.M., Bagot R.C., Purushothaman I., Issler O., Loh Y.-H.E., Leong T., Kiraly D.D. (2017). Early life stress confers lifelong stress susceptibility in mice via ventral tegmental area OTX2. Science.

[29] Walf A.A., Frye C.A. (2007). The use of the elevated plus maze as an assay of anxiety-related behavior in rodents. Nat. Protoc..

[30] Menard C., Pfau M.L., Hodes G.E., Kana V., Wang V.X., Bouchard S., Takahashi A., Flanigan M.E., Aleyasin H., LeClair K.B. (2017). Social stress induces neurovascular pathology promoting depression. Nat. Neurosci..

[31] Huang W., Hu W., Cai L., Zeng G., Fang W., Dai X., Ye Q., Chen X., Zhang J. (2020). Acetate supplementation produces antidepressant-like effect via enhanced histone acetylation. J. Affect. Disord..

[32] Deacon R.M.J. (2006). Assessing nest building in mice. Nat. Protoc..

[33] Luo Y.-J., Li Y.-D., Wang L., Yang S.-R., Yuan X.-S., Wang J., Cherasse Y., Lazarus M., Chen J.-F., Qu W.-M. (2018). Nucleus accumbens controls wakefulness by a subpopulation of neurons expressing dopamine D1 receptors. Nat. Commun..

[34] Nuzzo D., Amato A., Picone P., Terzo S., Galizzi G., Bonina F.P., Mulè F., Di Carlo M. (2018). A Natural dietary supplement with a combination of nutrients prevents neurodegeneration induced by a high fat diet in mice. Nutrients.

[35] Herzberg M.P., Gunnar M.R. (2019). Early life stress and brain function: Activity and connectivity associated with processing emotion and reward. NeuroImage.

[36] Cao P., Chen C., Liu A., Shan Q., Zhu X., Jia C., Peng X., Zhang M., Farzinpour Z., Zhou W. (2021). Early-life inflammation promotes depressive symptoms in adolescence via microglial engulfment of dendritic spines. Neuron.

[37] Li X., Gao T.-M. (2022). Epigenetic mechanism of depression after early life stress. Neurosci. Bull..

[38] Anda R.F., Felitti V.J., Bremner J.D., Walker J., Whitfield C.L., Perry B.D., Dube S.R., Giles W.H. (2005). The enduring effects of abuse and related adverse experiences in childhood. A convergence of evidence from neurobiology and epidemiology. Eur. Arch. Psychiatry Clin. Neurosci..

[39] Leuner B., Glasper E.R., Gould E. (2010). Parenting and plasticity. Trends Neurosci..

[40] Wang D., Levine J.L.S., Avila-Quintero V., Bloch M., Kaffman A. (2020). Systematic review and meta-analysis: Effects of maternal separation on anxiety-like behavior in rodents. Transl. Psychiatry.

[41] Enishi M., Ehorii-Hayashi N., Esasagawa T. (2014). Effects of early life adverse experiences on the brain: Implications from maternal separation models in rodents. Front. Neurosci..

[42] Nishi M. (2020). Effects of early-life stress on the brain and behaviors: Implications of early maternal separation in rodents. Int. J. Mol. Sci..

[43] Seok B.J., Jeon S., Lee J., Cho S.-J., Lee Y.J., Kim S.J. (2020). Effects of early trauma and recent stressors on depression, anxiety, and anger. Front. Psychiatry.

[44] Teissier A., Le Magueresse C., Olusakin J., da Costa B.L.S.A., De Stasi A.M., Bacci A., Kawasawa Y.I., Vaidya V.A., Gaspar P. (2019). Early-life stress impairs postnatal oligodendrogenesis and adult emotional behaviour through activity-dependent mechanisms. Mol. Psychiatry.

[45] Alves R.L., Portugal C., Summavielle T., Barbosa F., Magalhães A. (2019). Maternal separation effects on mother rodents’ behaviour: A systematic review. Neurosci. Biobehav. Rev..

[46] Reynolds K., Pietrzak R.H., El-Gabalawy R., Mackenzie C.S., Sareen J. (2015). Prevalence of psychiatric disorders in U.S. older adults: Findings from a nationally representative survey. World Psychiatry.

[47] Kessler R.C. (2003). Epidemiology of women and depression. J. Affect. Disord..

[48] Goodwill H.L., Nieves G.M., Gallo M., Lee H.I.S., Oyerinde E., Serre T., Bath K.G. (2018). Early life stress leads to sex differences in development of depressive-like outcomes in a mouse model. Neuropsychopharmacology.

[49] Bath K.G. (2020). Synthesizing views to understand sex differences in response to early life adversity. Trends Neurosci..

[50] Gracia-Rubio I., Moscoso-Castro M., Pozo O.J., Marcos J., Nadal R., Valverde O. (2015). Maternal separation induces neuroinflammation and long-lasting emotional alterations in mice. Prog. Neuro-Psychopharmacology Biol. Psychiatry.

[51] Han Y., Zhang L., Wang Q., Zhang D., Zhao Q., Zhang J., Xie L., Liu G., You Z. (2019). Minocycline inhibits microglial activation and alleviates depressive-like behaviors in male adolescent mice subjected to maternal separation. Psychoneuroendocrinology.

[52] Bonapersona V., Kentrop J., Van Lissa C., van der Veen R., Joëls M., Sarabdjitsingh R. (2019). The behavioral phenotype of early life adversity: A 3-level meta-analysis of rodent studies. Neurosci. Biobehav. Rev..

[53] O’Connor T.G., Willoughby M., Moynihan J.A., Messing S., Sefair A.V., Carnahan J., Yin X., Caserta M. (2019). Early childhood risk exposures and inflammation in early adolescence. Brain, Behav. Immun..

[54] Carpenter L.L.E., Gawuga C., Tyrka A.R., Lee J.K., Anderson G.M., Price L.H. (2010). Association between plasma IL-6 response to acute stress and early-life adversity in healthy adults. Neuropsychopharmacology.

[55] Réus G.Z., Fernandes G.C., de Moura A.B., Silva R.H., Darabas A.C., de Souza T.G., Abelaira H.M., Carneiro C., Wendhausen D., Michels M. (2017). Early life experience contributes to the developmental programming of depressive-like behaviour, neuroinflammation and oxidative stress. J. Psychiatr. Res..

[56] Prinz M., Jung S., Priller J. (2019). Microglia biology: One century of evolving concepts. Cell.

[57] Nayak D., Roth T.L., McGavern D.B. (2014). Microglia development and function. Annu. Rev. Immunol..

[58] Catale C., Bisicchia E., Carola V., Viscomi M.T. (2021). Early life stress exposure worsens adult remote microglia activation, neuronal death, and functional recovery after focal brain injury. Brain, Behav. Immun..

[59] Wang R., Wang W., Xu J., Liu D., Wu H., Qin X., Jiang H., Pan F. (2020). Jmjd3 is involved in the susceptibility to depression induced by maternal separation via enhancing the neuroinflammation in the prefrontal cortex and hippocampus of male rats. Exp. Neurol..

[60] Liu C., Dunkin D., Lai J., Song Y., Ceballos C., Benkov K., Li X.-M. (2015). Anti-inflammatory effects of ganoderma lucidum triterpenoid in human Crohnʼs disease associated with downregulation of NF-κB signaling. Inflamm. Bowel Dis..

[61] Jia Y., Zhang D., Yin H., Li H., Du J., Bao H. (2021). Ganoderic acid a attenuates LPS-induced neuroinflammation in BV2 microglia by activating farnesoid x receptor. Neurochem. Res..

[62] Ahmad R., Riaz M., Khan A., Aljamea A., Algheryafi M., Sewaket D., Alqathama A. (2021). *Ganoderma lucidum* (Reishi) an edible mushroom; a comprehensive and critical review of its nutritional, cosmeceutical, mycochemical, pharmacological, clinical, and toxicological properties. Phytotherapy Res..

[63] Liu J.Q., Lian C.L., Hu T.Y., Wang C.F., Xu Y., Xiao L., Liu Z.Q., Qiu S.Q., Cheng B.H. (2018). Two new farnesyl phenolic compounds with anti-inflammatory activities from Ganoderma duripora. Food Chem..

[64] Peng X.R., Liu J.Q., Wan L.S., Li X.N., Yan Y.X., Qiu M.H. (2014). Four new polycyclic meroterpenoids from Ganoderma cochlear. Org. Lett..

[65] Peng X.R., Liu J.Q., Wang C.F., Li X.Y., Shu Y., Zhou L., Qiu M.H. (2014). Hepatoprotective effects of triterpenoids from Ganoderma cochlear. J. Nat. Prod..

[66] National Center for Biotechnology Information. "PubChem Compound Summary for CID 471003" PubChem. https://pubchem.ncbi.nlm.nih.gov/compound/Ganoderic-acid-B.

[67] Yang M., Wang X., Guan S., Xia J., Sun J., Guo H., Guo D.A. (2007). Analysis of triterpenoids in ganoderma lucidum using liquid chromatography coupled with electrospray ionization mass spectrometry. J. Am. Soc. Mass Spectrom..

[68] National Center for Biotechnology Information. "PubChem Compound Summary for CID 21632955, methyl ganoderate B" PubChem. https://pubchem.ncbi.nlm.nih.gov/compound/methyl-ganoderate-B.

[69] National Center for Biotechnology Information. "PubChem Compound Summary for CID 20055990, CID 20055990" PubChem. https://pubchem.ncbi.nlm.nih.gov/compound/20055990.

[70] National Center for Biotechnology Information. "PubChem Compound Summary for CID 23247892, Lucidenic acid E" PubChem. https://pubchem.ncbi.nlm.nih.gov/compound/Lucidenic-acid-E..

[71] National Center for Biotechnology Information. "PubChem Compound Summary for CID 20055991, Ganoderic Acid J" PubChem. https://pubchem.ncbi.nlm.nih.gov/compound/Ganoderic-Acid-J..

[72] Chen L.X., Chen X.Q., Wang S.F., Bian Y., Zhao J., Li S.P. (2019). Analysis of triterpenoids in Ganoderma resinaceum using liquid chromatography coupled with electrospray ionization quadrupole - time - of - flight mass spectrometry. Int. J. Mass. Spectrom..

[73] National Center for Biotechnology Information. "PubChem Compound Summary for CID 14193982" PubChem. https://pubchem.ncbi.nlm.nih.gov/compound/14193982.

[74] National Center for Biotechnology Information. "PubChem Compound Summary for CID 102004760, CID 102004760" PubChem. https://pubchem.ncbi.nlm.nih.gov/compound/102004760..

[75] National Center for Biotechnology Information. "PubChem Compound Summary for CID 72728372, CID 72728372" PubChem. https://pubchem.ncbi.nlm.nih.gov/compound/72728372..

