# Supplementary material for: Ganoderma Lucidum Triterpenoids Improve Maternal Separation-Induced Anxiety- and Depression-like Behaviors in Mice by Mitigating Inflammation in the Periphery and Brain
Source: Nutrients. 2022 May 28;14(11):2268. doi: 10.3390/nu14112268 (PMC9182879; doi:10.3390/nu14112268)
Supplement: Supplementary file 1 [file nutrients-14-02268-s001.zip › nutrients-1722432-supplementary.pdf]

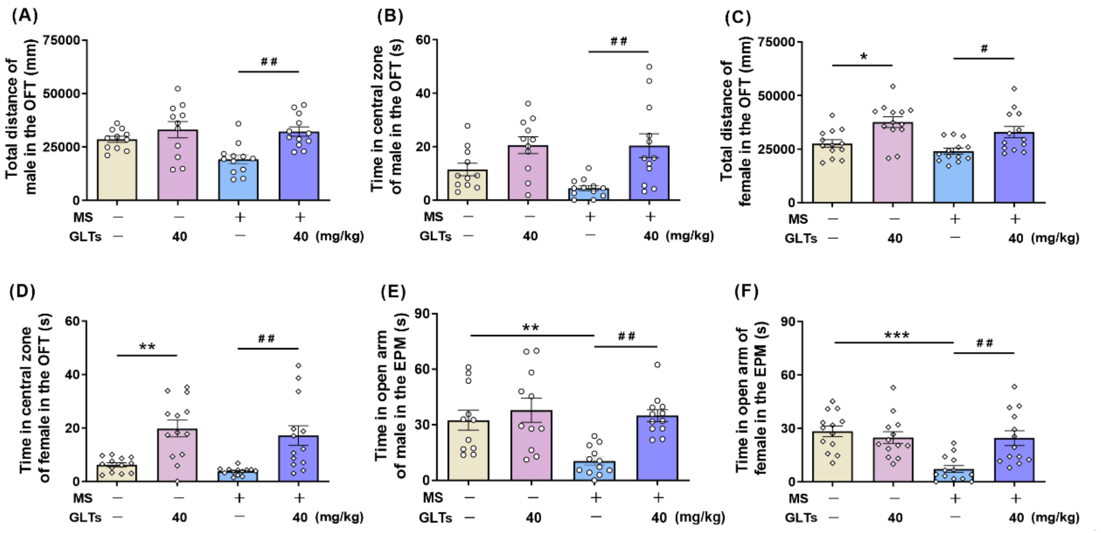

Figure S1. The similar effects of maternal separation and GLTs treatment on the anxiety-like behaviors of the male and female mice. (**A**) Total distance and (**B**) time spent in the central zone of the OFT in male mice. (**C**) Total distance and (**D**) time spent in the central zone of the OFT in female mice. (**E**) Time spent in the open arm of the EPM test in male mice. (**F**) Time spent in the open arm of the EPM test in male mice. Data are expressed as mean ± SEM. Number of male mice: Control + vehicle, n = 11; Control + GLTs (40 mg/kg), n = 11; MS + vehicle, n = 12; MS + GLTs (40 mg/kg), n = 12. Number of female mice: n = 13 / group. * $p < 0.05$, ** $p < 0.01$, *** $p < 0.001$, as compared with Control + vehicle group; # $p < 0.05$, ## $p < 0.01$, as compared with MS + vehicle.

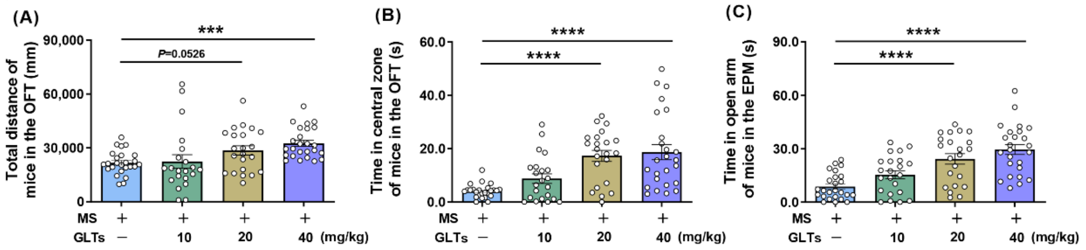

Figure S2. Effects of different doses of GLTs on anxiety-like behaviors of MS mice. (**A**) Total distance and (**B**) time spent in the central zone of the OFT. (**C**) Time spent in the open arm of the EPM. Data are expressed as mean ± SEM. Number of male mice: Control + vehicle, n = 25; MS + GLTs (10 mg/kg), n = 22; MS + GLTs (20 mg/kg), n = 22; MS + GLTs (40 mg/kg), n = 25. *** $p < 0.001$, **** $p < 0.0001$, as compared with MS + vehicle.

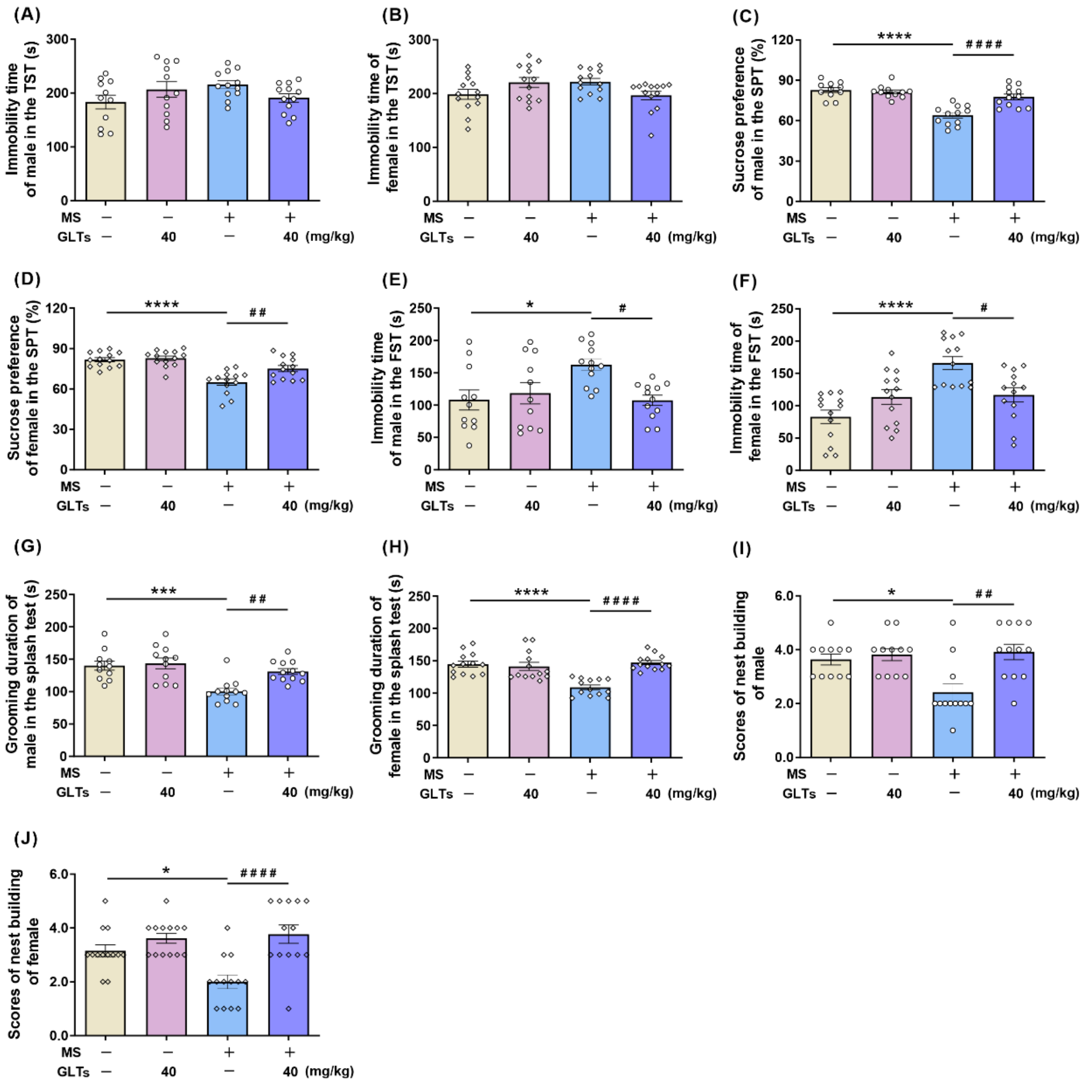

Figure S3. Similar effects of maternal separation and GLTs treatment on the depression-like behaviors in male and female mice. (**A**) Sucrose preference of male mice in the SPT. (**B**) Sucrose preference of female mice in the SPT. (**C**) Immobility time of male mice in the FST. (**D**) Immobility time of female mice in the FST. (**E**) Immobility time of male mice in the TST. (**F**) Immobility time of female mice in the TST. (**G**) Grooming duration of male mice in the splash test. (**H**) Grooming duration of female mice in the splash test. (**I**) Nest-building scores of male mice in the nest building test. (**J**) Nest-building scores of female mice in the nest building test. Data are expressed as mean ± SEM. Number of male mice: Control + vehicle, n = 11; Control + GLTs (40 mg/kg), n = 11; MS + vehicle, n = 12; MS + GLTs (40 mg/kg), n = 12. Number of female mice: n = 13 / group. * $p < 0.05$, *** $p < 0.001$, *** $p < 0.001$, as compared with Control + vehicle group; # $p < 0.05$, ## $p < 0.01$, #### $p < 0.0001$, as compared with MS + vehicle.

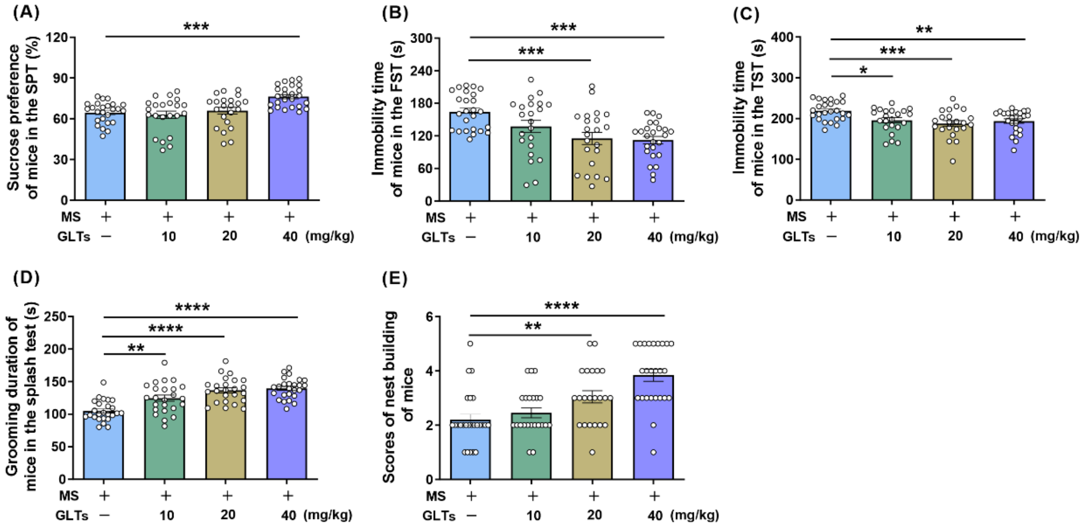

Figure S4. Effects of different doses of GLTs on depression-like behaviors in MS mice. (**A**) Sucrose preference of mice in the SPT. (**B**) Immobility time of mice in the FST. (**C**) Immobility time of mice in the TST. (**D**) Grooming duration of mice in the splash test. (**E**) Nest-building scores of mice in the nest building test. Data are expressed as mean ± SEM. Number of male mice: Control + vehicle, n = 25; MS + GLTs (10 mg/kg), n = 22; MS + GLTs (20 mg/kg), n = 22; MS + GLTs (40 mg/kg), n = 25. * $p < 0.05$, ** $p < 0.01$, *** $p < 0.001$, **** $p < 0.0001$, as compared with MS + vehicle.
